# Socioeconomic Status Correlates With Initial Patient-Reported Outcomes Measurement Information System-Pain Interference (PROMIS-PI) Scores but Not the Likelihood of Spine Surgery

**DOI:** 10.7759/cureus.57281

**Published:** 2024-03-30

**Authors:** Mark C Lawlor, Paul T Rubery, Caroline Thirukumaran, Gabriel Ramirez, Kathleen Fear

**Affiliations:** 1 Orthopaedic Surgery, University of Rochester, Rochester, USA; 2 Orthopaedic Surgery, UR Health Lab - University of Rochester Medical Center, Rochester, USA

**Keywords:** neck pain, back pain, worker's compensation, healthcare inequality, promis scores, cervical spine surgery, lumbar spine surgery, socioeconomic inequality

## Abstract

Objective

To explore how socioeconomic status and patient characteristics may be associated with initial self-reports of pain and determine if there was an increased association with undergoing spine surgery.

Methods

Patients at an academic center between 2015 and 2021 who completed the Patient-Reported Outcomes Measurement Information System-Pain Interference (PROMIS-PI) questionnaire were included. Multivariable linear regression models were used to determine the association between insurance type and patient factors with initial reports of pain. Multivariable logistic regression models were used to determine the association between PI and the likelihood of surgery in two time periods, three and 12 months.

Results

The study included 9,587 patients. The mean PROMIS-PI scores were 61.93 (SD 7.82) and 63.74 (SD 6.93) in the cervical and lumbar cohorts, respectively. Medicaid and Workers’ Compensation insurance patients reported higher pain scores compared to those with private insurance: Medicaid (cervical: 2.77, CI (1.76-3.79), p<0.001; lumbar (2.05, CI (1.52-2.59), p<0.001); Workers’ Compensation (cervical: 2.12, CI (0.96-3.27), p<0.001; lumbar: 1.51, CI (0.79-2.23), p<0.001). Black patients reported higher pain compared to White patients (cervical: 1.50, CI (0.44-2.55), p=0.01; lumbar: 1.51, CI (0.94-2.08), p<0.001). Higher PROMIS-PI scores were associated with a higher likelihood of surgery. There was no increased association of likelihood of surgery in Black, Medicaid, or Workers’ Compensation patients when controlling for pain severity.

Conclusion

Black patients and patients with Medicaid and Workers’ compensation insurance were likely to report higher pain scores. Higher initial pain scores were associated with an increased likelihood of surgery. However, despite increased pain scores, Black patients and those with Medicaid and Workers’ Compensation insurance did not have a higher likelihood of undergoing surgery.

## Introduction

Neck and back pain are two of the most prevalent complaints among adults and result in high healthcare costs [1-3]. In a resource-constrained healthcare system, accurate assessment of pain and function as well as the strategic deployment of those resources is critical. The Patient-Reported Outcomes Measurement Information System (PROMIS) offers a robust method to assess patient-reported outcomes (PROs) [4]. The PROMIS-Pain Interference (PI) score assesses pain interference with daily life. Prior PROMIS research demonstrated the ability to evaluate spine surgery outcomes utilizing PI [5-7].

Recent research has focused on socioeconomic status (SES) and potential healthcare disparities [8, 9]. The correlation between an individual’s SES, treatment options, and PROs is poorly understood. Crawford et al. revealed that Medicaid patients with lumbar spinal stenosis had systematically worse baseline PROs when compared to Medicare or private insurance [10]. It has also been shown that lower SES can predict worse outcomes in lumbar spine surgery [11].

Predicting which patients will respond to non-operative treatment and who may require surgery remains a challenge. Establishing whether social determinants influence the perception of pain may provide an opportunity for intervention and improved outcomes. No study has assessed the ability of PRO tools to predict the likelihood of spine surgery. Additionally, no study has analyzed whether a patient’s SES, insurance payor type, and other demographics correlate with an increased chance of surgery. An improved understanding of which patients are likely to undergo spine surgery based on information gathered from the initial encounter may allow for improved utilization of resources.

The study objectives were (i) identify spine patient characteristics associated with increased severity of self-reported pain at an initial encounter, (ii) assess whether patients with lower SES report greater initial pain or are more likely to undergo spine surgery and (iii) determine the correlation of pain severity as measured by the PROMIS-PI questionnaire with the likelihood of undergoing subsequent spine surgery. 

## Materials and methods

Data sources and study cohort

**Figure 1:**
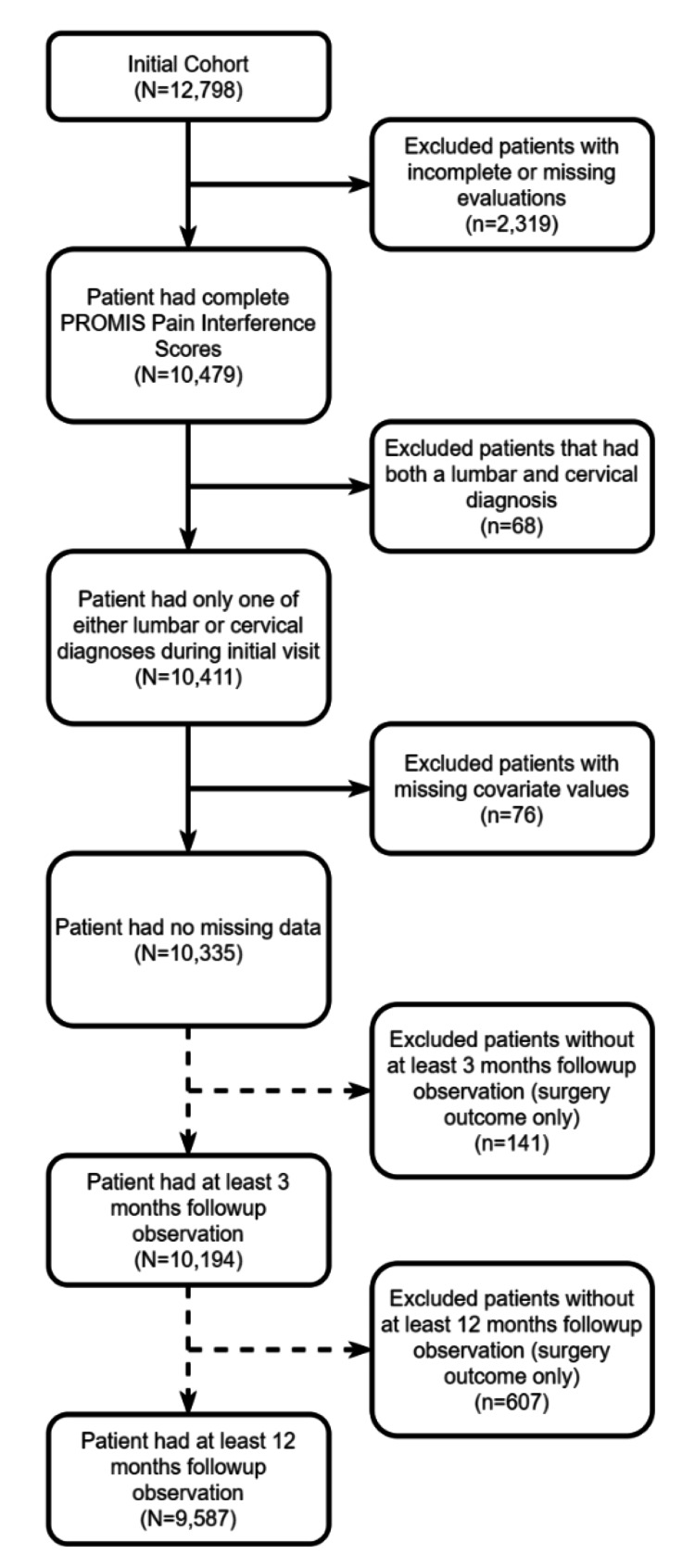
Cohort Inclusion/Exclusion Criteria

This study was approved by the Institutional Review Board (IRB #00000982). Informed consent was not required given the nature of the study and was deemed exempt by the Institutional Review Board. We used the International Classification of Diseases, 10th revision (ICD-10) diagnosis codes for common cervical or lumbar spine disorders including radiculopathy, spondylosis, and disc disease (Table 1) to retrospectively identify patients 18 years and older pursuing spine care at a large academic orthopedic spine clinic between January 2015 and January 2021. These patients completed PROMIS-PI (PROMIS-PI v1.1 or 1.2) computer adaptive tests on Apple iPads (Apple, Cupertino, CA) as part of standard care. A total of 12,798 patients met the ICD-10 diagnosis code inclusion criteria at their first outpatient encounter. We excluded patients missing PROMIS evaluations (n=2,319), patients with both a cervical and lumbar diagnosis (n=68), and patients with missing covariate values (n=76) (Figure 1). The final analytic cohort comprised initial encounters from 10,335 patients of which 2,428 patients were included in the cervical cohort and 7,983 patients in the lumbar cohort. For analyses examining the likelihood of surgery, we further excluded 141 patients and 607 patients, respectively, who did not have adequate follow-up data from the last three and 12 months. This resulted in cohorts of 10,194 and 9,587 patients from two time periods, three months and 12 months, respectively, for examining the likelihood of surgery following the initial outpatient encounter.

**Table 1:** List of International Classification of Disease, 10th Revision, Diagnosis Codes used for cohort identification

Code	Description
M4726	Other spondylosis with radiculopathy, lumbar region
M4727	Other spondylosis with radiculopathy, lumbosacral region
M5116	Intervertebral disc disorders with radiculopathy, lumbar region
M5117	Intervertebral disc disorders with radiculopathy, lumbosacral region
M5416	Radiculopathy, lumbar region
M5417	Radiculopathy, lumbosacral region
M4722	Other spondylosis with radiculopathy, cervical region
M4723	Other spondylosis with radiculopathy, cervicothoracic region
M5010	Cervical disc disorder with radiculopathy, unspecified cervical region
M5011	Cervical disc disorder with radiculopathy, high cervical region
M50121	Cervical disc disorder at C4-C5 level with radiculopathy
M50122	Cervical disc disorder at C5-C6 level with radiculopathy
M50123	Cervical disc disorder at C6-C7 level with radiculopathy
M5013	Cervical disc disorder with radiculopathy, cervicothoracic region
M5412	Radiculopathy, cervical region
M5413	Radiculopathy, cervicothoracic region

Outcomes

The outcomes of interest were continuous indicators of pain as measured by PROMIS-PI domains at the initial outpatient encounter. PROMIS domains are population normed to a mean T-score of 50 with a standard deviation of 10. Higher PROMIS-PI scores indicate more severe pain, while lower scores indicate a less severe pain level. To determine the likelihood of surgery, the outcomes of interest were separate binary indicators of whether or not a patient underwent surgery in three or 12 months following the initial outpatient encounter.

Key independent variables

Our key independent variable was the patient’s socioeconomic status as measured by a categorical indicator of the payor for the outpatient visit (Private, Medicare, Medicaid, Workers’ Compensation, other). Payor type has been utilized as a surrogate for socioeconomic status in multiple studies due to its consistent documentation in the electronic medical record system as a result of its link to billing [12-14]. Medicaid is a needs-based insurance that is based on state and federal guidelines regarding poverty indices [15]. In analyses examining the likelihood of surgery, additional key independent variables were continuous measures of pain severity as measured by PROMIS-PI scores.

Covariates

All multivariable models were controlled for continuous specifications of patient age and Elixhauser’s Comorbidity Index (ECI) [16] as well as categorical specifications of gender, race, ethnicity, smoking status, and alcohol consumption. We also controlled for the patient’s residence zip code characteristics including continuous specifications of the Area Deprivation Index (ADI), a validated composite measure of neighborhood socioeconomic deprivation [17-19], median household income, and percent with private insurance, percent unemployed, and percent with Bachelor’s degrees. We used the patient’s residence zip code and the Internal Revenue Service (IRS) Individual Income Tax Statistics database to obtain zip code-level median income [20].

Statistical analysis

We used means (and standard deviations) for continuous variables and numbers (and percentages) for categorical variables to describe patients in the cervical and lumbar cohorts. We first estimated multivariable linear regression models which examined the association between payor type (key independent variable) and the covariates listed above with the severity of pain (outcome) to determine the association of patient factors with pain reported at an initial outpatient visit. We next estimated multivariable logistic regression models to determine the association of pain and payor type (key independent variables) with the likelihood of surgery within three and 12 months of the initial visit (outcomes). All multivariable models controlled for the covariates listed above, and were separately estimated for the cervical and lumbar cohorts. To determine that our findings were robust to the specification of the pain variable, in the sensitivity analysis, we re-estimated the main models by defining pain as a binary variable (above and below median) in models examining pain as an outcome, and by defining pain as a categorical variable (quartile specification) in models examining the likelihood of surgery as an outcome. All data management and analysis were performed using STATA v17.1 (STATA Corp., College Station, TX). A p-value of <0.05 was indicative of statistical significance.

## Results

Descriptive statistics

Patient Characteristics

A total of 10,335 records were included by our inclusion and exclusion criteria (Figure 1). The mean age of the cervical cohort was 53.55 years (standard deviation [SD]: 12.95 years) and 57.45 years (SD: 16.13 years) for the lumbar cohort, and 55% of the patients in both cohorts were female (Table 2). Most patients were White (cervical: 84.64%, n=2044/2415; lumbar: 86.55%, n=6855/7920) and not of Hispanic ethnicity (cervical: 96.15%, n=2322/2415; lumbar: 96.50%, n=7643/7920). The main payor group for both cohorts was private payor (cervical: 55.73%, n=1346/2415; lumbar: 46.39%, n=3674/7920). Medicaid insured 12.17% (294/2415) of the patients in the cervical cohort and 11.05% (875/7920) in the lumbar cohort. Median household income was $63,180 (SD: $40,000) in the cervical cohort and $65,820 (SD: $42,110) in the lumbar cohort. The mean PROMIS-PI score at the first outpatient visit was 61.93 (SD 7.82) in the cervical cohort and 63.74 (SD 6.93) in the lumbar cohort.

**Table 2:** Characteristics and pain metrics for patients with encounters for cervical or lumbar conditions from 2015 to 2021 N: number, %: column percentage, SD: standard deviation, PROMIS-PI: Patient-Reported Outcomes Measurement Information System-Pain Interference *comorbidities were identified using Elixhauser’s algorithm.

Characteristics	Cervical cohort	Lumbar cohort
Patients (N)	2,415	7,920
Age in years; Mean (SD)	53.55 (12.95)	57.45 (16.13)
Sex; N (%)		
Male	1,096 (45.38)	3,585 (45.27)
Female	1,319 (54.62)	4,335 (54.73)
Race; N (%)		
White	2,044 (84.64)	6,855 (86.55)
Black	259 (10.72)	718 (9.07)
Other/unknown	112 (4.64)	347 (4.38)
Ethnicity; N (%)		
Hispanic	93 (3.85)	277 (3.50)
Non-Hispanic	2,322 (96.15)	7,643 (96.50)
Comorbidities;^*^ N (%)		
None	2,408 (99.71)	7,863 (99.28)
One or more	7 (0.29)	57 (0.72)
Smoking status; N (%)		
Never	1,236 (51.18)	4,076 (51.46)
Current	410 (16.98)	1,125 (14.20)
Former/unknown	769 (31.84)	2,719 (34.33)
Alcohol consumption; N (%)		
Never	844 (34.95)	2,931 (37.01)
Current	1,239 (51.30)	4,107 (51.86)
Former/unknown	332 (13.75)	882 (11.14)
Payor; N (%)		
Private	1,346 (55.73)	3,674 (46.39)
Medicare	464 (19.21)	2,824 (35.66)
Medicaid	294 (12.17)	875 (11.05)
Workers’ Compensation	185 (7.66)	373 (4.71)
Other/Unknown	126 (5.22)	174 (2.20)
Characteristics of zip code of patient’s residence		
Zip code; N	149	226
Area Deprivation Index; mean (SD)	7.15 (1.51)	7.03 (1.54)
Median income (in $1,000); mean (SD)	63.18 (40.00)	65.82 (42.11)
Percent with private insurance; mean (SD)	71.97 (2.98)	72.08 (3.05)
Percent unemployed; mean (SD)	6.38 (1.10)	6.36 (1.07)
Percent with bachelor’s degree; mean (SD)	31.72 (7.60)	31.78 (7.46)
Pain metrics		
PROMIS-PI; mean (SD)	61.93 (7.82)	63.74 (6.93)

Multivariate analysis

Self-Reported Pain Interference at the Initial Outpatient Encounter

After controlling for relevant covariates and compared to private payor patients, Medicaid patients in the cervical cohort reported 2.77 points higher PROMIS-PI scores (95% Confidence Interval [CI]: 1.76 to 3.79, p<0.001) and 2.05 points higher PROMIS-PI scores (CI: 1.52 to 2.59, p<0.001) in the lumbar cohort at initial encounter (Table 3). Furthermore, Workers’ Compensation patients reported 2.12 points higher and 1.51 points higher PROMIS-PI scores in the cervical and lumbar cohorts, respectively (cervical: CI: 0.96 to 3.27, p<0.001; lumbar: CI: 0.79 to 2.23, p<0.001). Patients covered through “other insurance”, which included self-pay, motor vehicle accident (MVA), or unknown status also reported higher pain.

**Table 3:** Beta estimates from multivariable linear regression models examining the association between patient risk factors and pain metrics * p<0.05, ** p<0.01, *** p<0.001 A CI of 95% was set. N: number, CI: confidence interval; SD: standard deviation, PROMIS-PI: Patient-Reported Outcomes Measurement Information System-Pain Interference; Ref: reference

	Cervical cohort, PROMIS-PI	Lumbar cohort, PROMIS-PI
Payor		
Private	Ref	Ref
Medicaid	2.77*** (1.76, 3.79)	2.05*** (1.52, 2.59)
Medicare	0.75 (-0.18, 1.69)	0.21 (-0.22, 0.65)
Workers’ Compensation	2.12*** (0.96, 3.27)	1.51*** (0.79, 2.23)
Other/Unknown	4.12*** (2.75, 5.48)	1.39** (0.37, 2.41)
Age	-0.15 (-0.42, 0.13)	0.15* (0.02, 0.28)
Sex		
Female	Ref	Ref
Male	-1.34*** (-1.94, -0.73)	-0.53*** (-0.83, -0.23)
Race		
White	Ref	Ref
Black	1.50** (0.44, 2.55)	1.51*** (0.94, 2.08)
Other/Unknown	1.08 (-0.49, 2.64)	0.15 (-0.63, 0.94)
Ethnicity		
Non-Hispanic	Ref	Ref
Hispanic	1.20 (-0.51, 2.91)	1.23** (0.35, 2.12)
Elixhauser’s comorbidities	5.53* (0.03, 11.02)	0.61 (-1.09, 2.31)
Smoking status		
Never	Ref	Ref
Current	3.17*** (2.29, 4.04)	2.89*** (2.43, 3.36)
Former/Unknown	1.31*** (0.62, 1.99)	0.97*** (0.63, 1.30)
Alcohol consumption		
Never	Ref	Ref
Current	-1.02** (-1.69, -0.36)	-0.90*** (-1.23, -0.57)
Former/Unknown	-1.42** (-2.38, -0.45)	-0.71** (-1.23, -0.20)
Zip code characteristics		
Area Deprivation Index	0.22 (-0.06, 0.50)	0.22 (0.08, 0.36)
Median income (in $1,000)	0.00 (-0.01, 0.01)	-0.01 (-0.01, 0.00)
Percent with private insurance	-0.05 (-0.17, 0.07)	-0.01 (-0.07, 0.06)
Percent unemployed	0.05 (-0.24, 0.33)	0.09 (-0.06, 0.25)
Percent with bachelor’s degree	0.00 (-0.05, 0.04)	-0.01 (-0.03, 0.02)
N	2,415	7,920

Compared to White patients, statistically significant higher pain scores were found reported by Black patients in both cohorts (cervical: 1.50, CI: 0.44 to 2.55, p: 0.01; lumbar: 1.51, CI: 0.94 to 2.08, p<0.001). Males reported lesser pain than female patients (cervical: -1.34, CI: -1.94 to -0.73, p<0.001; lumbar: -0.53, CI: -0.83 to -0.23, p<0.001). Compared to patients who had never smoked, higher pain scores were reported by current smokers in both cohorts (cervical: 3.17, CI: 2.29 to 4.04, p<0.001; lumbar: 2.89, CI: 2.43 to 3.36, p<0.001), and by former smokers or those with an unknown smoking history (cervical: 1.31, CI: 0.62 to 1.99, p<0.001; lumbar: 0.97, CI: 0.63 to 1.30, p<0.001). The findings of the sensitivity analysis were consistent with the findings from the main analysis.

Likelihood of Surgery

On multivariable analysis, the odds of surgery within 3 and 12 months of the initial encounter for patients in the cervical cohort increased by 6% (three months' adjusted odds ratio [AOR]: 1.06, CI: 1.03 to 1.09, p<0.001; 12 months' AOR: 1.06, CI: 1.03 to 1.08, p<0.001) with each unit increase in the PROMIS-PI score (Table 4). Similarly, in the lumbar cohort, the odds of surgery increased by 6% to 7% (three months' AOR: 1.07, CI: 1.06 to 1.09, p<0.001; 12 months' AOR: 1.06, CI: 1.05 to 1.08, p<0.001) with each unit increase in the PROMIS-PI score.

**Table 4:** Odds ratios from multivariable logistic regression models examining the association between pain metrics (specified as continuous variables) and the likelihood of surgery within three or 12 months of the initial encounter * p<0.05, ** p<0.01, *** p<0.001 A CI of 95% was set. N: number, CI: confidence interval; SD: standard deviation, PROMIS-PI: Patient-Reported Outcomes Measurement Information System-Pain Interference; Ref: reference

Parameters	Cervical surgery (PROMIS-PI)		Lumbar surgery (PROMIS-PI)	
	3 months	12 months	3 months	12 months
PROMIS-PI T-Score	1.06*** (1.03-1.09)	1.06*** (1.03-1.08)	1.07*** (1.06-1.09)	1.06*** (1.05-1.08)
Payor				
Private	Ref	Ref	Ref	Ref
Medicaid	0.88 (0.47, 1.62)	1.10 (0.67, 1.78)	0.87 (0.61, 1.23)	0.91 (0.69, 1.21)
Medicare	0.51* (0.27, 0.97)	0.76 (0.45, 1.28)	1.37* (1.03, 1.83)	1.22 (0.97, 1.54)
Workers’ Compensation	0.32* (0.11, 0.91)	0.51 (0.25, 1.07)	0.76 (0.45, 1.27)	0.76 (0.50, 1.15)
Other	0.51 (0.20, 1.34)	0.56 (0.25, 1.28)	0.69 (0.32, 1.51)	0.82 (0.45, 1.49)
Gender				
Female	Ref	Ref	Ref	Ref
Male	1.47 (0.98, 2.19)	1.52* (1.09, 2.11)	1.41*** (1.15, 1.72)	1.40*** (1.19, 1.64)
Race				
White	Ref	Ref	Ref	Ref
Black	0.92 (0.45, 1.86)	1.06 (0.60, 1.86)	0.75 (0.51, 1.12)	0.87 (0.64, 1.19)
Other	1.02 (0.36, 2.89)	1.54 (0.71, 3.38)	0.91 (0.52, 1.59)	0.72 (0.44, 1.16)
Ethnicity				
Non-Hispanic	Ref	Ref	Ref	Ref
Hispanic	0.80 (0.25, 2.55)	0.69 (0.28, 1.74)	0.63 (0.32, 1.23)	1.10 (0.68, 1.78)
Age (in decades)	1.25* (1.03, 1.53)	1.14 (0.97, 1.34)	0.90* (0.82, 0.98)	0.97 (0.90, 1.04)
Elixhauser’s comorbidities	--	--	0.29 (0.04, 2.09)	0.33 (0.08, 1.36)
Smoking status				
Never	Ref	Ref	Ref	Ref
Current	1.78* (1.06, 3.00)	1.69* (1.10, 2.59)	1.21 (0.91, 1.62)	1.30* (1.03, 1.64)
Former or unknown	0.93 (0.58, 1.51)	0.88 (0.59, 1.31)	1.00 (0.79, 1.26)	1.06 (0.88, 1.27)
Alcohol consumption				
Never	Ref	Ref	Ref	Ref
Current	1.00 (0.65, 1.55)	1.13 (0.79, 1.63)	1.01 (0.81, 1.26)	1.08 (0.91, 1.30)
Former or unknown	0.78 (0.39, 1.55)	0.92 (0.53, 1.60)	1.01 (0.72, 1.42)	1.19 (0.91, 1.56)
Zip code characteristics				
Area Deprivation Index	0.89 (0.73, 1.09)	0.92 (0.78, 1.07)	1.00 (0.91, 1.10)	1.00 (0.93, 1.08)
Median income (in $1,000)	0.99* (0.98, 1.00)	0.99* (0.99, 1.00)	1.00 (0.99, 1.00)	1.00 (1.00, 1.00)
Percent with private insurance	0.92* (0.86, 0.98)	0.91*** (0.86, 0.96)	0.97* (0.93, 1.00)	0.96** (0.93, 0.98)
Percent unemployed	0.73*** (0.60, 0.88)	0.72*** (0.62, 0.84)	0.74*** (0.67, 0.81)	0.72*** (0.66, 0.79)
Percent with bachelor’s degree	1.02 (0.99, 1.06)	1.01 (0.98, 1.04)	1.00 (0.98, 1.02)	1.00 (0.99, 1.02)
N	2,377	2,239	7,817	7,348

Notably, the odds of surgery were not significantly different for patients insured by Medicaid compared to private payor patients (cervical [three months] AOR: 0.88, CI: 0.47 to 1.62, p: 0.68; cervical [12 months] AOR: 1.10, CI: 0.67 to 1.78, p: 0.71; Lumbar [three months] AOR: 0.87, CI: 0.61 to 1.23, p: 0.43; lumbar [12 months]: AOR: 0.91, CI: 0.69 to 1.21, p: 0.53). The odds of cervical surgery at three months were 68% lower for Workers’ Compensation patients compared to privately insured patients (AOR: 0.32, CI: 0.11 to 0.91, p: 0.03).

Overall, the odds of surgery were not significantly different for Black patients compared to White patients in both cervical and lumbar cohorts. We also noted higher odds of lumbar surgery for male patients (lumbar [three months] AOR: 1.41, CI: 1.15 to 1.72, p<0.001; lumbar [12 months] AOR: 1.40, CI: 1.19 to 1.64, p<0.001); and cervical surgery in patients who reported as current smokers (cervical [three months] AOR: 1.78, CI: 1.06 to 3.00, p: 0.03; cervical [12 months]: AOR: 1.69, CI: 1.10 to 2.59, p: 0.01). 

**Figure 2:**
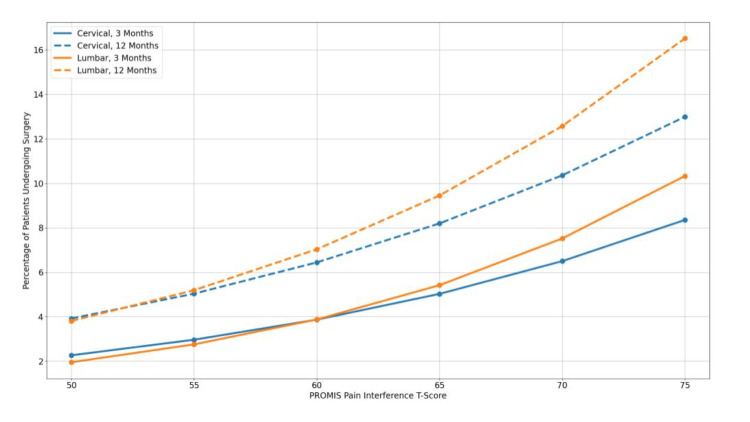
Adjusted percentages of undergoing cervical or lumbar surgeries within three or 12 months of the initial encounters. Estimates derived from regression models presented in Table 3.

Patients ​in the cervical cohort with the lowest PROMIS-PI score of 50 had a 2.26% chance of surgery at three months vs 3.92% at 12 months of the initial encounter (Figure 2). For the highest PROMIS-PI score of 70, patients had a 6.50% chance of surgery at three months and 10.36% at 12 months. The lumbar cohort showed the same trend with patients with PROMIS-PI of 50 having a 1.95% chance of surgery at three months vs 3.81% at 12 months. Patients with PROMIS-PI scores of 70 had a 7.52% chance of surgery at three months and 12.57% at 12 months. 

On sensitivity analysis, patients with pain scores above the median, i.e. quartiles 3 and 4, had significantly greater odds of surgery relative to the lower quartile patients which is consistent with the main analysis showing increased likelihood of surgery with increasing pain score.

## Discussion

The impact of social determinants of health on pain in spine conditions, including SES, is under-analyzed. A more complete understanding of a patient’s SES may improve outcomes. We sought to explore how SES and patient characteristics may impact initial self-reports of pain and determine if there was an increased association with undergoing spine surgery.

In our study, we determined an association of Medicaid and Workers’ Compensation payor status with higher reported pain scores as measured by the PROMIS-PI questionnaire at initial visits for cervical or lumbar conditions. Additionally, we determined an association of higher pain scores with the increased likelihood of surgery within three and 12 months. However, despite increased initial pain scores in patients with Medicaid or Workers’ Compensation insurance, we did not find a statistically significant increase in their likelihood of surgery when compared to patients with private insurance. Similarly, Black patients reported higher initial pain scores compared to White patients but did not have a higher likelihood of undergoing surgery. Current smoking status was the only variable that correlated with both higher initial pain and increased likelihood of surgery. This is the first study to analyze the association of patient demographics, insurance status, and the likelihood of undergoing spine surgery. Additionally, this is the first study to report quantitative increases in the likelihood of surgery based on initial self-reports of PI.

Our study shows that patients from lower socioeconomic strata, those with Workers’ Compensation or Medicaid insurance, who are Black, and who smoke present with worse pain. We found that worse initial pain scores were typically associated with an increased likelihood of undergoing spine surgery. However, when controlling for payor status, and when comparing Black and White patients there was no difference in the likelihood of surgery. There are several potential explanations for this. Surgeons may not use payor status or objective measures of pain as their main determinants for offering surgery. Surgeons may have a higher threshold for offering surgery to patients with Medicaid or Workers’ Compensation insurance or for those who are Black and may require either more non-surgical treatment or have more significant levels of pain. Interestingly, our data suggests that increased PI does increase the odds of surgery among smokers, a group where surgical outcomes are known to be compromised.

Challenges in access to care for Workers’ Compensation and Medicaid patients may result in increased PI at initial presentation. Surgeon biases regarding pain perception in Medicaid and Workers’ Compensation patients and resultant altered surgical thresholds may explain our observed lack of increased likelihood of surgery despite increased PI. Surgeons may prefer to delay surgery or trial non-operative management for longer in these patients when compared to other payor types, leading to care disparities. Alternatively, patients with lower SES may not elect to proceed surgically due to a lack of familiarity with spine surgery or mistrust in the healthcare system, a well-documented theory in the total joint arthroplasty literature [21-23]. Higher initial PI not accompanied by increased surgical likelihood in Black, Medicaid, and Workers’ Compensation patients may indicate a previously unidentified disparity in spinal surgery.

As healthcare reimbursement moves towards outcome-based payment structures, a more comprehensive model should be instituted. One solution that has been suggested by others is utilizing a relative change model as opposed to an absolute number when calculating change in outcome measures [4, 16]. Instead of utilizing benchmark postoperative outcome metrics, which statistically are lower for patients with lower SES, a model comparing improvement from preoperative scores could be instituted. Providers who treat large numbers of patients with lower SES may see differing pre- and post-operative PI scores compared to a provider with a mainly private payor practice. Our findings support adjusting payment structures to reflect the SES of patients to avoid unjustly penalizing providers caring for patients with lower SES [24, 25]. 

This study has limitations. First, only patients presenting to a single academic medical center spine clinic were included in our analysis. The population was predominately White with private or Medicare insurance. However, our large sample ensures an analysis with robust results that are likely more generalizable than smaller studies in the literature. Second, we used the insurance payor as a surrogate for SES which inherently has some confounding bias. However, qualifying for need-based insurance payor status based on state and federal standards is a valid surrogate for socioeconomic status. Our analyses were adjusted for potential confounders though it is possible that other significant factors exist that we were unable to control for. Finally, we are unable to comment on how PROMIS-PI scores may have changed following surgical intervention in those patients who underwent surgery; exploring this is a future aim of our research.

## Conclusions

In conclusion, we found that patients with Medicaid or Workers’ Compensation insurance and Black patients reported higher initial PI and that higher initial PI scores were associated with an increased likelihood of undergoing spine surgery for either cervical or lumbar disease. However, when controlling for the severity of pain, these patients did not have an increased likelihood of surgery compared to White patients or those covered by private insurance. These findings suggest patients from lower SES present with either worse PI, or worse perception of their pain, and yet they do not have an increased likelihood of surgery. Further research is needed to understand the underlying causes of these findings and to identify strategies to improve healthcare access.
